# Loneliness and barriers to medication adherence in older adults: A multicenter cross-sectional study

**DOI:** 10.1371/journal.pone.0352895

**Published:** 2026-11-13

**Authors:** Bruna de Freitas Brazzolotto, Karina Bertoldi, Rafaella Stradiotto Bernardelli, Ana Clara Borges Monti, Camila Tonet, Lucas Sperka Furlan, Giovanna Nogarolli Faria, Gabriela Bonilha Nogueira, Fabiana Luiza Hornung, Karen Barros Parron Fernandes, Cristina Pellegrino Baena

**Affiliations:** 1 Health Sciences Graduate Program (PPGCS), Pontifical Catholic University of Paraná (PUCPR), Curitiba, Paraná, Brazil; 2 School of Medicine and Health Sciences, Pontifical Catholic University of Paraná (PUCPR), Curitiba, Paraná, Brazil; 3 School of Medicine and Health Sciences, Pontifical Catholic University of Paraná (PUCPR), Londrina, Paraná, Brazil; Faculty of Pharmacy - Istinye University, TÜRKIYE

## Abstract

Population aging is linked to the increase of chronic diseases and use of multiple medications. Consequently, medication non-adherence constitutes a global challenge. Therefore, we aimed to analyze the factors associated with the implementation phase of non-adherence. A multicenter cross-sectional study was conducted with older adults (age ≥ 60) in outpatient clinics of the public health system in Brazil. Medication adherence was assessed by the Measure of Adherence to Treatment (MAT), with non-adherence defined by a score below 5 points. Data were obtained through structured interviews, including the evaluation of loneliness (UCLA-BR scale) and barriers (adapted BMQ), and analyzed using multivariable logistic regression (Wald, p < 0.05). The study included 597 patients (*M* age = 71.3, *SD* = 7.2, 56.3% female) with comorbidities (cardiovascular 84.4%, endocrinological 82.4%). Non-adherence was present in 22.4% (*n* = 134) and was independently associated with recruitment site (Curitiba vs. Londrina: *OR* = 1.878, 95% CI [1.119, 3.153], *p* = 0.017), belief-related barriers (*OR* = 2.259, 95% CI [1.434, 3.557], *p* < 0.001), and practical barriers (*OR* = 2.662, 95% CI [1.361, 5.207], *p* = 0.004). A graded association emerged between loneliness and non-adherence: each additional point in the UCLA-BR score increased the odds of non-adherence by 3.0% (*OR* = 1.030, 95% CI [1.013, 1.047], *p* < 0.001). The findings reveal implementation non-adherence as a sociobehavioral phenomenon. Loneliness, practical and belief barriers, and recruitment site were independently associated with higher odds of non-adherence and could be potential targets for adherence interventions in an older population.

## Introduction

Global aging is a striking factor observed in the 21st century associated with the decline in birth and mortality rates, resulting in an exponential increase in the older adult population [1,2]. It is estimated that by 2050, the number of older people in the world will double, reaching approximately 2.1 billion individuals, and more than half of global deaths are expected to occur over the age of 80 years old [3–5].With the growing population aging, there is also a significant increase in chronic non-communicable diseases, which require continuous pharmacological treatment and are associated with higher rates of polypharmacy and therapeutic complexity [6]. These factors are markedly known to reduce medication adherence which, consequently, contributes to increased morbidity and mortality, worsening of quality of life, and overburdening of health services [7].

The WHO (2003) defines adherence to therapy as “the extent to which a person’s behavior – taking medication, following a diet, and/or executing lifestyle changes, corresponds with agreed recommendations from a health care provider” [8]. Through an expert consensus, Vrijens et al. (2012) proposed a uniform taxonomy since there was a lack of terminological standardization. Medication adherence was thus defined as “The process by which patients take their medications as prescribed, composed of initiation, implementation and discontinuation” [9].

By this definition, initiation is the process by which the patient takes the first dose of the medication as prescribed. Implementation refers to the degree of correlation between the prescription regimen and the patient's actual use. The period between the first and last medication doses is defined as persistence. Finally, the last dose used characterizes discontinuation. Medication non-adherence is therefore characterized by inadequacy at any stage of the adherence process: delay or non-initiation, sub-implementation of the therapeutic regimen, or early discontinuation [9].

Behaviorally, these failures can also be classified into two strands: intentional and unintentional non-adherence. Intentionality, as the term suggests, is the patient's active decision to alter or interrupt treatment, whereas unintentional non-adherence occurs passively, resulting from forgetfulness, lack of understanding of the guidelines, or everyday practical barriers [10].

In an attempt to explain this process, several theoretical models have been proposed. The WHO (2003) categorized the main determinants of adherence into five interdependent dimensions: factors related to the health system, the patient, the therapy, the condition, and socioeconomic aspects (World Health Organization, 2003). Subsequently, Unni and Bae (2022) proposed a hierarchical model of fundamental psychological parameters for the consolidation of adherence behavior. In the pyramidal structure, health literacy forms the foundation, followed by beliefs about the disease and treatment, and self-efficacy, which culminate at the apex of medication adherence [8,11].

From the perspective of optimizing public resources, medication non-adherence significantly increases health system expenditures: an additional $ 949 to $ 52,341 per patient per year. These costs are related to hospitalizations, a higher demand for complementary exams, escalation of therapeutic plans, and rehabilitation [12].

Studies suggest that medication non-adherence levels reach close to 50%, proving to be a prevalent factor in the population [8,13–15]. In Brazil, medication adherence ranges from 5.4% to 88.24% depending on the method used [16,17]. However, determinants of medication non-adherence are still underexplored in the literature. The systematic review by Afkhami et al. (2025) demonstrated that less than 1.5% of studies explore the factors reducing adherence in older adults [7]. Within this context, the need to understand the specific factors associated of each population emerge.

### Objective

To analyze the factors associated with medication non-adherence of an older adult population in outpatient follow-up by the public health system in southern Brazil.

## Methods

### Study design

This is an observational, multicenter, and cross-sectional study, conducted with patients aged 60 or older, at two tertiary outpatient sites affiliated with the Pontifical Catholic University of Paraná, located in Curitiba and Londrina, Paraná, Brazil. Both sites provide outpatient care within the Brazilian public health system and included patients followed in cardiology, internal medicine, family and community medicine, endocrinology, gastroenterology, and other outpatient specialties.. Data collection was conducted from August 5, 2024, to June 27, 2025,

The study received ethical approval from the Research Ethics Committee of Pontifical Catholic University of Paraná (the proposing institution) on 04/05/2024, under approval protocol number 6.772.702 (ID CAAE: 78740924.7.0000.0020). Administrative authorizations were also obtained from participating centers prior to data collection. All participants provided written informed consent prior to enrollment. Data were anonymized before analysis and handled in accordance with confidentiality standards. All study procedures were conducted in accordance with the protocol approved by the Research Ethics Committee and with the principles of the Declaration of Helsinki (8th revision, 2024)..

This study was conducted in compliance with the guidelines of the Strengthening the Reporting of Observational Studies in Epidemiology (STROBE), Risk of Bias Tools for Medication Adherence Research (RoBIAS and RoBOAS) and the ESPACOMP Medication Adherence Reporting Guideline (EMERGE) [18–20].

### Participants

The study was conducted at tertiary outpatient clinics of the public health system associated with a Brazilian university. The facility encompasses 20 clinical and 36 surgical specialties, with clinical consultations representing approximately 45% of the total volume. A consecutive sampling strategy was adopted, in which all patients who met the eligibility criteria and attended outpatient consultations during the data collection period (August 2024 to June 2025) were recruited. No a priori sample-size calculation was performed; therefore, the study sample size was determined by the number of eligible participants recruited during this period.

Patients aged 60 years or older, of both sexes, who voluntarily agreed to participate and signed the written informed consent form were considered eligible for the study. Patients were excluded if they had a diagnosis of dementia, recent hospitalization (less than 10 days prior to data collection), were not using any continuous medications, and/or did not have an electronic medical record demonstrating regular follow-up in the outpatient clinics included in the study.

### Data collection

Data collection was performed through structured interviews, analysis of electronic medical records, and application of validated questionnaires, which were conducted by researchers trained by experts. The collected variables encompassed socioeconomic factors – such as age, sex, education, marital status, and average family income; behavioral factors, such as smoking history, alcohol consumption, drug addiction, use of corrective lenses, reported hearing impairment, weekly physical activity, daily sedentary time, living alone, and loneliness severity; and health factors, such as comorbidities, cognitive assessment by the Montreal Cognitive Assessment (MoCA), functional dependence for Basic Activities of Daily Living (BADL) by the Katz Index, and muscle strength through dynamometry measurement. Data regarding the racial and ethnic distribution of the participants were not collected in this study.

Loneliness severity was operationally defined and measured using the Brazilian version of the UCLA Loneliness Scale (UCLA-BR), validated for the Portuguese language. The scale consists of 20 items scored on a 4-point Likert scale (0 = never to 3 = frequently), yielding a total score ranging from 0 to 60 points. Participants were categorized into four loneliness severity levels based on validated cut-off values: minimal loneliness (0–22 points), mild loneliness (23–35 points), moderate loneliness (36–47 points), and severe loneliness (48–60 points).

### Evaluation of medication adherence

Medication adherence was measured using the validated Measure of Adherence to Treatment (MAT) instrument, which evaluates the implementation phase of pharmacological treatment by analyzing the patient's daily medication-taking behavior. Consisting of seven questions related to medication adherence, the answers are obtained on a Likert-type scale ranging from 1 (always) to 6 (never). The result is calculated by the average responses (sum of all values divided by the number of items). Participants with scores lower than 5 points were considered non-adherent [21–24]. In the current sample, the MAT demonstrated adequate internal consistency, with Cronbach’s alpha of  .816.

Additionally, the adapted Brief Medication Questionnaire (BMQ) was administered to identify barriers to treatment adherence. Two sections were utilized. Belief-related barriers (Section 2) were assessed using the screening item, “Do any of your medications cause problems for you?” Affirmative responses indicated the presence of belief-related barriers, categorized dichotomously (presence vs. absence), and were followed by a qualitative specification of the implicated medications and perceived adverse effects.

Recall and practical barriers (Section 3) were evaluated by investigating five physical and logistical challenges related to medication management: opening or closing containers, reading package labels, remembering to take all doses, obtaining refills, and swallowing multiple pills simultaneously. Responses were rated on a Likert scale from “very difficult” to “no problem” and subsequently dichotomized as presence (any reported difficulty) or absence (no difficulty reported).

Section 1 of the BMQ was excluded to prevent data redundancy during interviews. Comprehensive pharmacological profiles, including drug names, dosages, and daily schedules, were systematically collected during clinical characterization using a triple-verification method (patient self-report, active medical prescriptions, and electronic health records). This approach streamlined the assessment while providing a more accurate and verified medication record than short-term recall.

Conceptually, the MAT quantifies the behavioral implementation outcome (i.e., medication-taking frequency), whereas the BMQ subscales capture the underlying operational and physical barriers contributing to non-adherence.

### Statistical analysis

For statistical analysis, categorical variables are presented as absolute and percentage frequencies; quantitative variables with a normal distribution are presented by mean and standard deviation; and quantitative variables without a normal distribution are presented by median and interquartile range. Normality was assessed using the Kolmogorov-Smirnov test and visual inspection of the box plot.

Categorical variables were compared between the adherent and non-adherent groups by the chi-square test or Fisher's exact test, when appropriate. Quantitative variables with normal distribution were compared between the two groups using the Student's T-test for independent samples, and when the assumption of data normality was not met, the Mann-Whitney test was used.

Variables considered potentially relevant to medication non-adherence were selected for evaluation based on their theoretical or clinical relationship with the outcome. These included age, sex, outpatient clinic specialty, education, marital status, living alone, monthly family income, number of comorbidities, cognitive impairment, use of corrective lenses, hearing impairment, physical inactivity, sedentary time, smoking history, alcohol consumption history, history of falls in the previous 12 months, sit-to-stand test performance, dynapenia, functional dependence in basic activities of daily living, loneliness (UCLA-BR loneliness score), belief-related barriers, practical barriers, use of adherence aids, and number of pills taken per day. Because of the multicenter design, recruitment site (Curitiba or Londrina) was also evaluated as a potential factor associated with medication non-adherence. This prespecification determined the variables evaluated in the univariable analyses and was independent of their statistical associations with the outcome.

Each variable was first examined separately using univariable binary logistic regression, with medication non-adherence (MAT < 5) as the dependent variable. Odds ratios (ORs) and their corresponding 95% confidence intervals (95% CIs) were estimated. Variables with a *p* value <0.200 in the univariable analysis were selected as candidates for the multivariable logistic regression model.

Before multivariable model fitting, multicollinearity among candidate variables was assessed using the variance inflation factor (VIF). VIF values <2.5 were considered to indicate no relevant multicollinearity. All candidate variables meeting the selection criterion and showing no evidence of relevant multicollinearity were entered simultaneously into the initial multivariable logistic regression model (Enter method). A backward Wald procedure was subsequently applied to obtain the final adjusted model.

Two-way interactions were also examined before fitting the final multivariable model based on theoretical plausibility and, for recruitment site, on observed differences in participant characteristics between centers. To this end, separate logistic regression models containing both main effects and their interaction term were fitted. An interaction was considered statistically significant when the Wald test *p* value for the interaction term was < 0.05. Only statistically significant interaction terms were to be included in the multivariable model.

The relationship between the number of outcome events and model complexity was examined by considering the number of medication non-adherence events relative to the number of estimated predictor parameters in both the initial and final multivariable models. For categorical predictors, the number of estimated indicator parameters, rather than the number of variables alone, was considered when assessing model complexity.

Model calibration was assessed using the Hosmer–Lemeshow goodness-of-fit test, whereas discriminatory performance was evaluated using the area under the receiver operating characteristic curve (AUC) and its 95% CI.

No missing data were imputed. Regression analyses were conducted using complete-case analysis (listwise deletion), whereby participants with missing data for any variable included in a given model were excluded from that analysis. The number of observations included in each analysis was reported, and the extent of missing data for individual variables was specified in the corresponding table footnotes.

All statistical tests were two-sided, and a *p* value <0.05 was considered statistically significant, except for the *p* < 0.200 criterion used to select candidate variables for multivariable modeling. Analyses were performed using IBM SPSS Statistics, version 28.0 (IBM Corp., Armonk, NY, USA).

## Results

Initially, 656 participants aged 60 years or older, of both sexes, and regularly followed up at the included clinics were approached. After applying the exclusion criteria, 597 patients were considered eligible and included in the analysis (Fig 1).

**Fig 1:**
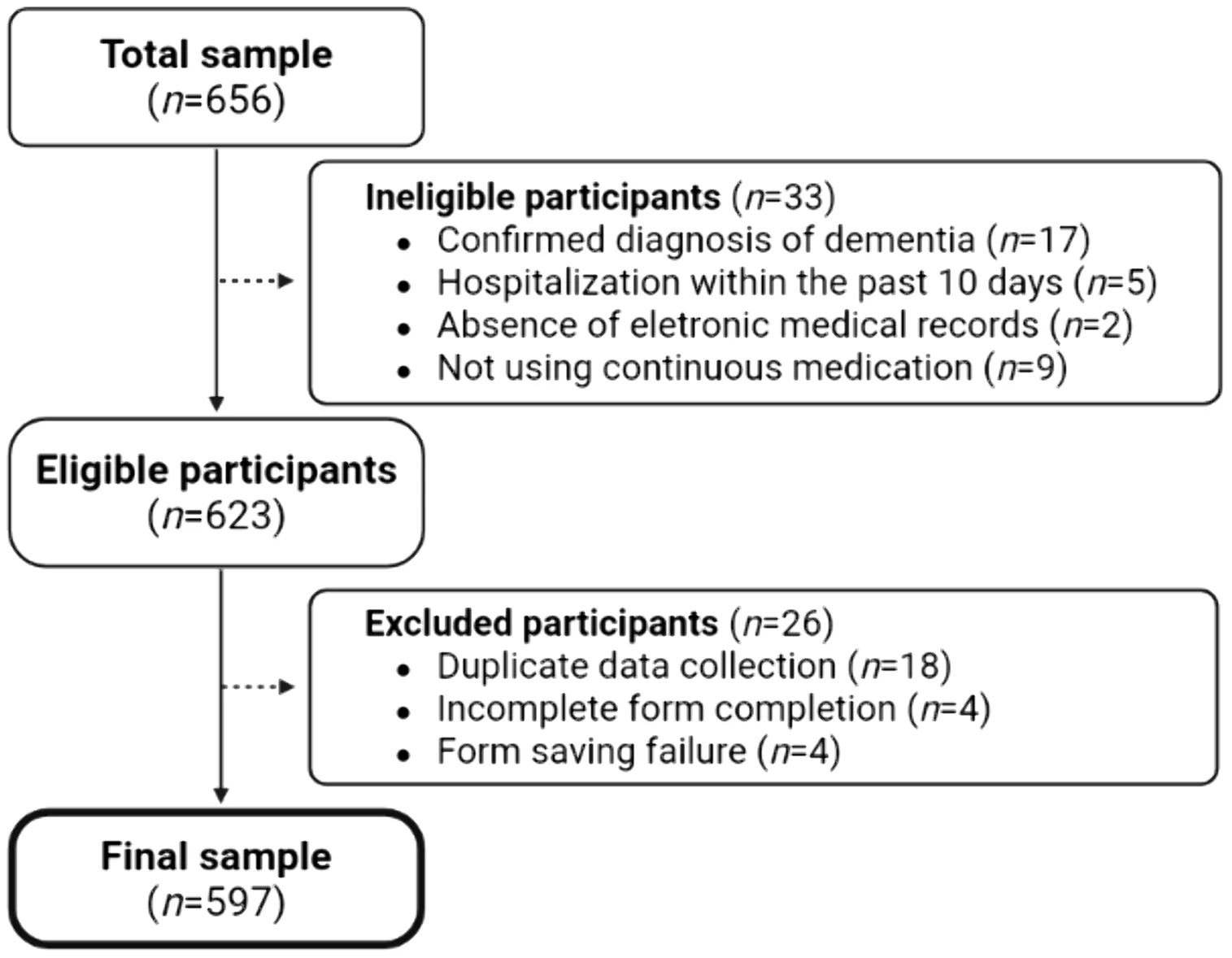
Flowchart.

Data collection was conducted in two cities in the state of Paraná, Brazil. A total of 387 (64.8%) participants were recruited in Curitiba and 210 (35.2%) in Londrina. Participants from the two recruitment sites differed significantly with respect to age, sex, education, outpatient clinic specialty, number of comorbidities, muscle status, sedentary time, functional dependence for basic activities of daily living, loneliness score, number of pills taken per day, and the presence of belief-related barriers (S1 Table).

The final sample consisted of 597 patients, with a mean age of 71.3 ± 7.2 years, mostly female (56.3%), married or in a stable union (54.8%), with low education (76.4%), and a monthly family income between 1 and 3 minimum wages (72.4%). Medication non-adherence was present in 22.4% (*n* = 134) of the sample (Table 1).

**Table 1:** Main characteristics.

Variable	Total Sample *n* = 597	Adherent (MAT ≥ 5)*n* = 463	Non- adherent (MAT < 5)*n* = 134	*p* value
**Sociodemographic characteristics**				
Age	71.3 ± 7.2	71.3 ± 7.4	71.3 ± 6.7	.747^§^
Age group				.714^#^
60 to 69 years	294 (49.2)	231 (49.9)	63 (47)	
70 to 79 years	227 (38)	172 (37.1)	55 (41)	
80 years or older	76 (12.7)	60 (13)	16 (11.9)	
Gender				.843*
Female	336 (56.3)	262 (56.6)	74 (55.2)	
Male	261 (43.7)	201 (43.4)	60 (44.8)	
Education				.192^#^
Low (≤ 7 years of study)	456 (76.4)	346 (74.7)	110 (82.1)	
Medium (8–11 years of study)	94 (15.7)	77 (16.6)	17 (12.7)	
High (≥ 12 years of study)	47 (7.9)	40 (8.6)	7 (5.2)	
Marital status				.404^#^
Single	53 (8.9)	38 (8.2)	15 (11.2)	
Married or stable union	327 (54.8)	251 (54.2)	76 (56.7)	
Widowed	132 (22.1)	103 (22.2)	29 (21.6)	
Separated or divorced	85 (14.2)	71 (15.3)	14 (10.4)	
Monthly family income (in Brazilian minimum wages)^a,b^				.486^#^
Less than 1	46 (8.1)	36 (8.1)	10 (8.1)	
Between 1 and 3	411 (72.4)	325 (73.2)	86 (69.4)	
Between 3 and 6	91 (16)	66 (14.9)	25 (20.2)	
7 or more	20 (3.5)	17 (3.8)	3 (2.4)	
Outpatient clinics				0.015^#^
Cardiology	301 (50.4)	219 (47.3)	82 (61.2)	
Internal Medicine or Family and Community Medicine	32 (5.4)	24 (5.2)	8 (6)	
Endocrinology	87 (14.6)	71 (15.3)	16 (11.9)	
Gastroenterology	60 (10.1)	46 (9.9)	14 (10.4)	
Others	117 (19.6)	103 (22.2)	14 (10.4)	
Recruitment site				<.001*
Londrina, Paraná, Brazil	210 (35.2)	185 (40.0)	25 (18.7)	
Curitiba, Paraná, Brazil	387 (64.8)	278 (60.0)	109 (81.3)	
**Comorbidities**				
Number of comorbidities	6 (4567–8)	5 (456–7)	6 (4567–8)	.216*
Cardiovascular diseases	504 (84.4)	386 (83.4)	118 (88.1)	.224*
Endocrinological diseases	492 (82.4)	382 (82.5)	110 (82.1)	.898*
Gastrointestinal disorders	237 (39.7)	186 (40.2)	51 (38.1)	.689*
Neurological disorders	100 (16.8)	85 (18.4)	15 (11.2)	0.065*
Psychiatric disorders	193 (32.3)	153 (33)	40 (29.9)	.530*
Urinary tract disorders	122 (20.4)	93 (20.1)	29 (21.6)	.716*
Rheumatological disorders and/or chronic pain	254 (42.5)	194 (41.9)	60 (44.8)	.554*
Infectious diseases	28 (4.7)	23 (5)	5 (3.7)	.649*
Pulmonary diseases	103 (17.3)	78 (16.8)	25 (18.7)	.606*
History of neoplasia	68 (11.4)	52 (11.2)	16 (11.9)	.877*
Vascular diseases	24 [4]	18 (3.9)	6 (4.5)	.803*
**Functional characteristics**				
Maximal handgrip strength (kgf)^c,d^	21.5 (16– 30)	22 (16–30)	21 (14.5–28)	.081^‡^
Muscle status by sex-specific cut-offs, according to dynamometry. ^c,d^				.005*
Eutrophic	374 (63.2)	301 (65.4)	73 (55.3)	
Dynapenia	218 (36.8)	159 (34.6)	59 (44.7)	
Sit-to-stand test ^e^	8 (5–10)	8 (5–11)	8 (5– 10)	.501^‡^
Functional dependence for BADL^g^	165 (27.6)	119 (25.7)	46 (34.3)	.062*
Degree of functional dependence for BADLs^g^				.041^#^
Independent	432 (72.4)	344 (74.3)	88 (65.7)	
Mild dependence (1–2 functions)	137 (22.9)	95 (20.5)	42 (31.3)	
Moderate dependence (3–4 functions)	14 (2.3)	11 (2.4)	3 (2.2)	
Severe dependence (≥ 5 functions)	14 (2.3)	13 (2.8)	1 (0.7)	
History of falls in the past 12 months	169 (28.3)	125 (27)	44 (32.8)	.193*
Physically inactive (< 150 min/week)	476 (79.7)	364 (78.6)	112 (83.6)	.244*
Sedentary time (hours)^f^	4 (2–6)	4 (2–6)	4 (2–6)	.505^‡^
**Biopsychosocial characteristics**				
Sensory Impairment				
Use of corrective lenses	476 (79.7)	374 (80.8)	102 (76.1)	.272*
Hearing impairment reported by others	206 (34.5)	156 (33.7)	50 (37.3)	.470*
Substance Use				
Smoking history (current or former)	283 (47.4)	214 (46.2)	69 (51.5)	.326*
Alcohol consumption history (current or former)	209 (35)	156 (33.7)	53 (39.6)	.218*
Living alone	106 (17.8)	85 (18.4)	21 (15.7)	.523*
UCLA-BR loneliness score, range 0–60	6 []2–15)	5 (2–13)	12 (4 - 23)	<.001^‡^
Presence of loneliness	92 (15.4)	55 (11.9)	37 (27.6)	<.001*
Loneliness severity (UCLA-BR scale)				<0.001
Minimal loneliness (0–22 points)	505 (84.6)	408 (88.1)	97 (72.4)	
Mild loneliness (23–35 points)	53 (8.9)	35 (7.6)	18 (13.4)	
Moderate loneliness (36–47 points)	29 (4.9)	16 (3.5)	13 (9.7)	
Severe loneliness (48–60 points)	10 (1.7)	4 (0.9)	6 (4.5)	
Cognitive classification (education-adjusted MoCA scores)				.845
Preserved cognition	316 (52.9)	244 (52.7)	72 (53.7)	
Cognitive impairment	281 (47.1)	219 (47.3)	62 (46.3)	
**Continuous-use medications**				
Number of pills per day	8 (5–12)	8 (5–12)	9 (6–13)	.067^‡^
Presence of belief-related barriers	136 (22.8)	82 (17.7)	54 (40.3)	<.001
Presence of one or more practical barriers	472 (79.1)	350 (75.6)	122 (91)	<.001
Opening or closing the medication bottle	111 (18.6)	73 (15.8)	38 (28.4)	<.001
Reading the print on the bottle	346 (57.9)	263 (56.8)	83 (61.9)	.211
Remembering to take the pills	196 (32.8)	113 (24.4)	83 (61.9)	<.001
Getting your refills in time	209 (35)	138 (29.8)	71 (53)	<.001
Taking so many pills at the same time	121 (20.3)	70 (15.1)	51 (38.1)	<.001
Use of adherence aids ^h,i^	393 (66.8)	305 (67)	88 (66.2)	.971

Categorical variables are expressed as absolute frequencies (percentages). Age is reported as mean ± standard deviation (SD), and other quantitative variables are expressed as median (*Md*) and interquartile range (IQR). ^a^Brazilian minimum wages (2025): 1518.00 Brazilian Real/BRL = 287.72 US Dollar/USD. ^b^19 missing data points in the adherent group and 10 in the non-adherent group. ^c^ Handgrip strength was assessed based on three consecutive measurements in both hands. The highest value was used for statistical analysis. Values below 16 kgf for women and 27 kgf for men were defined as dynapenia. ^d^3 missing data points in the adherent group and 2 in the non-adherent group. ^e^Performance in the sit-to-stand test was determined by the total number of full repetitions completed in a 30-second interval. Patients who were unable to perform the test were assigned a score of zero. ^f^Sedentary time was estimated based on self-reported daily sitting hours per day. ^g^The basic activities of daily living (BADLs) assessed by the Katz Index include: bathing, dressing, toileting, transferring, continence, and feeding. ^h^The following adherence aids were assessed: pill organizers, administration by a caregiver, use of alarms, drawings on medication packaging, drawings on medical prescriptions, use of medication schedules, storage in separate containers, checking prescriptions before intake, and organizing medications in order of administration. ^i^8 missing data points in the adherent group and 1 in the non-adherent group. *Significance based on Fisher's exact test. ^#^Significance based on the Chi-square test. ^§^Significance based on the Student's t-test for independent samples. ^‡^Significance based on the non-parametric Mann-Whitney U test.

A minority of patients reported having some belief barrier regarding medications (*n* = 136; 22.8%). When cited, the main beliefs were related to the presence of side effects (*n* = 102; 17.1%), doubts regarding medication efficacy (*n* = 15; 2.51%), and skepticism about the need for treatment (*n* = 8; 1.34%), fear of dependency or clinical worsening (*n* = 7; 1.17%).

When compared to adherent patients, non-adherent patients had a greater presence of belief barriers (40.3% vs. 17.7%, *p* < 0.001) and practical barriers (91% vs. 75.6%, *p* < 0.001), higher UCLA-BR loneliness scores (median 12 [IQR 4–23] vs. 5 [IQR 2–13], *p* < 0.001), had a higher degree of dependence for BADLs (34.3% vs. 25.7%, *p* = 0.062), were more dynapenic (44.7% vs. 34.6%, *p* = 0.005) and more frequently recruited from the Curitiba site (81.3% vs. 60.0%, *p* < 0.001) (Table 1).

Variables considered potentially relevant to medication non-adherence were prespecified a priori based on their theoretical relationship with the outcome. These variables were individually examined using univariable binary logistic regression, with medication non-adherence as the dependent variable (Table 2).

**Table 2:** Univariable logistic regression analysis of factors associated with medication non-adherence.

Variable	Valid n	OR (95% CI) for medication non-adherence (MAT < 5)*	*p* value
Age (years)	597	1.000 (.974–1.027)	.976
Male gender	597	1.057 (.718–1.556)	.779
Curitiba, Paraná, Brazil as recruitment site (Ref.: Londrina, Paraná, Brazil)	597	2.901 (1.808–4.655)	<.001
Outpatient clinics (ref.: cardiology)	597		
Internal Medicine or Family and Community Medicine		.890 (.385–2.061)	.786
Endocrinology		.602 (.331–1.095)	.097
Gastroenterology		.813 (.424–1.557)	.532
Others		.363 (.197 – .670)	.001
Education (ref.: high [≥ 12 years of study])	597		
Low (≤ 7 years of study)		1.262 (.483–3.293)	.635
Medium (8–11 years of study)		1.817 (.791–4.171)	.159
Marital status (ref.: married)	597		
Widowed		.930 (.572–1.511)	.769
Separated or divorced		.651 (.348–1.220)	.181
Single		1.304 (.680–2.498)	.424
Living alone	597	.826 (.490–1.393)	.474
Monthly family income (in Brazilian minimum wages) (ref: less than 1)	568		
Between 1 and 3		.953 (.455–1.996)	.898
Between 3 and 6		1.364 (.590–3.153)	.468
7 or more		.635 (.155–2.611)	.529
Number of comorbidities	597	1.040 (.969–1.115)	.281
Cognitive impairment, classificated by education-adjusted MoCA scores	597	.959 (.653–1.411)	.833
Use of corrective lenses	597	.759 (.479–1.201)	.238
Hearing impairment reported by others	597	1.171 (.786–1.747)	.438
Physically inactive (< 150 min/week)	597	1.385 (.833–2.302)	.209
Sedentary time (hours)	597	1.048 (.995–1.103)	.078
Smoking history (current or former)	597	1.235 (.841–1.815)	.282
Alcohol consumption history (current or former)	597	1.288 (.866–1.914)	.211
History of falls in the past 12 months	597	1.322 (.873–2.002)	.187
Sit-to-stand test		.984 (.944–1.026)	.441
Dynapenia	592	1.530 (1.033–2.267)	.034
Functional dependence for BADL		1.511 (1.000–2.284)	.050
UCLA-BR loneliness score, range 0–60	597	1.042 (1.027–1.058)	<.001
Presence of belief-related barriers	597	3.136 (2.061–4.772)	<.001
Presence of one or more practical barriers	597	3.282 (1.749–6.161)	<.001
Use of adherence aids	597	1.040 (.691–1.565)	.852
Number of pills per day	597	1.036 (.998–1.075)	.062

Abreviations: BADL, Basic Activities of Daily Living; UCLA-BR, Brazilian version of the UCLA Loneliness Scale; Ref., reference variable. * Odds ratios (OR) and 95% confidence intervals (95% CI) from univariable binary logistic regression models for the odds of medication non-adherence. § Wald test p-value; p < .05 indicates statistical significance.

Variables with a p value <0.200 in the univariable logistic regression analysis were selected as candidates for the multivariable model. Multicollinearity among these candidate variables was assessed before model fitting using the variance inflation factor (VIF), and no relevant multicollinearity was identified, with all VIF values <2.5 (S2 Table). These variables were subsequently entered into the multivariable logistic regression model (Table 3).

**Table 3:** Multivariable logistic regression analysis of factors associated with medication non-adherence.

Variable	OR (95% CI) for medication non-adherence (MAT < 5)*	*p* value
**INITIAL FULL MODEL (ENTER)**		
Curitiba, Paraná, Brazil as recruitment site (Ref.: Londrina, Paraná, Brazil)	1.818 (.916 - 3.606)	.087
Outpatient clinics (ref.: Cardiology)		
Internal Medicine or Family and Community Medicine	1.305 (.516 - 3.300)	.574
Endocrinology	.755 (.39 - 1.462)	.404
Gastroenterology	.813 (.389 - 1.696)	.580
Others	.771 (.346 - 1.717)	.524
Education (ref.: high; ≥ 12 years of study)		
Low; ≤ 7 years of study)	1.469 (.526 - 4.1)	.463
Medium; 8–11 years of study)	1.718 (.699 - 4.221)	.238
Marital status (ref.: married)		
Widowed	.75 (.439 - 1.283)	.294
Separated or divorced	.554 (.28 - 1.095)	.089
Single	1.17 (.581 - 2.359)	.660
Sedentary time (hours)	1.016 (.959 - 1.076)	.585
History of falls in the past 12 months	.726 (.446 - 1.181)	.197
Dynapenia	.917 (.582 - 1.444)	.709
Functional dependence for BADL	1.049 (.641 - 1.716)	.849
UCLA-BR loneliness score, range 0–60	1.032 (1.015 - 1.05)	<.001
Presence of belief-related barriers	2.386 (1.492 - 3.814)	<.001
Presence of one or more practical barriers	2.849 (1.433 - 5.663)	.003
Number of pills per day	1.001 (.957 - 1.047)	.958
n^#^	592	
Hosmer-Lemeshow test (Chi-square; degrees of freedom)	1.956; 8	.204
Model discrimination (AUC [95% CI])‡	.738 (.692 − .783)	<.001
**FINAL ADJUSTED MODEL (STEPWISE BACKWARD WALD)**		
Curitiba, Paraná, Brazil as recruitment site (Ref.: Londrina, Paraná, Brazil)	1.878 (1.119 - 3.153)	.017
UCLA-BR loneliness score, range 0–60	1.030 (1.013 - 1.047)	<.001
Presence of belief-related barriers	2.259 (1.434 - 3.557)	<.001
Presence of one or more practical barriers	2.662 (1.361 - 5.207)	.004
n^#^	592	
Hosmer-Lemeshow test (Chi-square; degrees of freedom)¬	13.748; 8	.089
AUC (95% CI)‡	.720 (.673 − .766)	<.001

Abreviations: BADL, Basic Activities of Daily Living; AUC, area under the Receiver Operating Characteristic (ROC) curve; UCLA-BR, Brazilian version of the UCLA Loneliness Scale; Ref., reference variable. * Odds ratios (OR) and 95% confidence intervals (95% CI) from multivariable binary logistic regression model for the odds of medication non-adherence. § Wald test p-value; p < 0.05 indicates statistical significance. # Number of cases included in the model. ¬ The goodness-of-fit of the multivariable model, expressed by Hosmer-Lemeshow test. ‡ The discriminatory performance of the multivariable model, expressed by the area under the ROC curve (AUC) and its 95% confidence interval for the model's predicted probability of the outcome.

In the results of the final adjusted multivariable model, recruitment site, loneliness, belief-related barriers, and practical barriers remained independently associated with medication non-adherence. A graded association was observed between loneliness severity and non-adherence, with progressively higher UCLA-BR loneliness scores associated with increased odds of non-adherence. Participants recruited in Curitiba also had higher odds of non-adherence than those recruited in Londrina, while the presence of belief-related and practical barriers was associated with approximately two- to three-fold higher odds of non-adherence. The final model showed adequate goodness-of-fit and acceptable discriminatory performance (Hosmer–Lemeshow p = .089; AUC = .720, 95% CI  .673–.766) (Table 3).

Among the 592 participants included in the multivariable analysis, 132 presented medication non-adherence (22.3%). The initial model included 12 candidate variables, corresponding to 18 estimated predictor parameters, yielding approximately 7.3 outcome events per parameter. Following backward Wald selection, the final model retained four predictor parameters, corresponding to approximately 33 outcome events per estimated parameter.

Before multivariable modeling, prespecified two-way interactions were examined based on theoretical plausibility. Separate logistic regression models were fitted for each pair of variables, including both main effects and their interaction term. These analyses included the interaction between UCLA-BR loneliness score and marital status; pairwise interactions among sedentary time, history of falls in the previous 12 months, dynapenia, and functional dependence in basic activities of daily living; and interactions between recruitment site and variables that differed significantly between the two centers, including outpatient clinic specialty, education, dynapenia, sedentary time, functional dependence in basic activities of daily living, loneliness score, number of pills taken per day, and belief-related barriers. No statistically significant interaction terms were identified; therefore, no interaction terms were added to the multivariable model.

## Discussion

The findings of this study reveal that medication non-adherence during the implementation phase among older adults followed by the public health system in southern Brazil is a multidimensional sociobehavioral phenomenon shaped by the convergence of cognitive-attitudinal, functional, and psychosocial factors. Four independent factors were associated with higher odds of non-adherence in the adjusted model: the recruitment site, the presence of belief-related barriers, the presence of practical barriers, and the perceived severity of loneliness. Notably, loneliness did not act merely as a binary factor associated with medication non-adherence but exhibited a graded association with non-adherence, underscoring that its influence deepens progressively as social disconnection intensifies and pointing to a dynamic process rather than a threshold effect. Together, these findings question reductionist approaches to medication adherence and support a framework in which the biopsychosocial context of aging is central to understanding and ultimately addressing failures in treatment implementation.

This result builds on previous ones, indicating the importance of social roles in health-related outcomes [7,25–29]. By definition, loneliness consists of the perceived discrepancy between social expectations and the actual degree of social connection, which differs from social isolation, social support, or even depression, although they can be interrelated [28,30]. Older adult patients usually go through a transition phase of their social role due to lifestyle changes related to retirement, independence of children, death of a partner and friends and a lower level of social interaction, with these changes being associated with a greater perception of loneliness [27,28].

Several theoretical models try to explain the role of loneliness in chronic diseases [25,27,28]. Hawkley and Cacioppo (2010) state that the perception of loneliness increases vigilance for social threats, which, in turn, alters psychological and physiological processes. Unconsciously, through this state of hypervigilance, lonely individuals become more prone to confirmation and memory biases, expecting negative social outcomes and more easily recalling adverse experiences [25].

Moreover, the model describes a ‘regulatory loop of loneliness’ where loneliness reduces self-regulation capacity, negatively impacting behavior and lifestyle [25]. Although this study does not directly address medication non-adherence, the model provides grounds to hypothesize that loneliness may be associated with intentional non-adherence through belief barriers (e.g., concerns about medication safety) and lower self-regulation (e.g., the habit of non-adherence).

In 2020, Lu et al. developed and validated an integrated theoretical model to explain the mechanisms linking social isolation and loneliness to medication non-adherence in older adults. The study concluded that social isolation exerts a negative indirect impact on adherence through a serial mediation model: social isolation observed during aging reduces social support, which in turn intensifies the perception of loneliness, resulting in medication non-adherence. In this context, loneliness does not act in isolation but emerges as the final component in a chain of events exacerbated by the lack of social support [27]. Yu et al. (2024) confirmed this serial mediation model through a cohort of 797 Chinese older adults with chronic diseases [28].

Based on this, loneliness is a key factor in non-adherence. Our study, built on previous studies that examined the association between loneliness and non-adherence [7,28], highlighted a less-explored characteristic: a graded association between these two variables. When modeled continuously, each one-point increase in the UCLA-BR score was associated with a 3.0% increase in the adjusted odds of non-adherence. This finding supports a graded association across the loneliness continuum.

As far as could be identified, there are no studies that explicitly describe a graded association between different levels of loneliness and medication non-adherence in older adults with chronic diseases. The existing literature demonstrates only linear associations or mediations involving loneliness and adherence. Therefore, to the best of our knowledge, this is the first study to describe such a gradual association.

The presence of barriers also proved to be associated with higher odds of non-adherence. Horne and Weinman (1999) were the first authors to describe concern beliefs and necessity beliefs as necessary factors for the adherence process. For them, medication adherence is driven by the tension between these two belief models, with the “necessity-concern difference” a robust predictor of adherence, having a greater impact than sociodemographic factors and the number of medications [31]. This association was subsequently confirmed by other authors. However, patients with multiple comorbidities present a peculiarity: in this group, ensuring that necessity beliefs exceed concerns is not sufficient to determine adherence, indicating a more complex, multifactorial process [32].

One possible explanation relates to the form of non-adherence. Intentional non-adherence is strongly characterized by the weighing between the cost vs. benefit of the treatment, as described by Horne and Weinman. On the other hand, unintentional non-adherence (such as forgetting or being careless with prescribed doses) is unconsciously related to concerns. This suggests that forgetfulness is not merely a memory deficit, and that interventions based only on visual reminders or alarms may fail if the patient's concerns are not addressed [10].

The current study, however, focused on analyzing the association between concern belief barriers and general non-adherence, demonstrating that, regardless of the evaluation of necessity beliefs, older adult patients who possess concern beliefs had approximately 2.3 -fold higher odds of being non-adherent. This result supports the hierarchical model of adherence proposed by Unni and Bae (2022), which identifies beliefs about treatment as an important pillar of medication adherence [11].

Due to the inherent factors of aging, older adult patients exhibit greater susceptibility to functional decline, which affects basic and instrumental activities of daily living, potentially leading to a bidirectional relationship with non-adherence. In this context, worsening functionality reduces the patient's ability to coordinate medications, generating non-adherence [33]. This, in turn, worsens comorbidities and leads to consequent functional impairment [34], creating an important feedback loop. In our study, this vulnerability was evidenced quantitatively by greater functional dependence in basic activities of daily living among non-adherent patients. It is exactly this decline that translates into the main practical barriers to medication handling, as assessed by the adapted BMQ questionnaire.

The reported visual difficulty with reading labels was the only limitation that did not differ statistically between the adherent and non-adherent groups. This finding can be attributed to the homogeneity of the sample, since almost 80% of the patients had visual alterations requiring corrective lenses, which limits the statistical power to detect differences between the groups. The result, however, is consistent with McCann et al.’s (2012) findings, which compared medication adherence between older adult patients with and without visual impairment in a case-control study. The study concluded that, although there was no difference in adherence between groups, patients with visual difficulty needed more help to handle medications [35].

As demonstrated in the literature, other practical barriers were more prevalent among non-adherent patients: difficulty in opening and closing packages [35–38], swallowing all pills simultaneously [39], obtaining medications [39,40], and remembering to take medications [33,36,38–40]. Despite the relevance of the results, the Cochrane Library systematic review found a paucity of high-quality evidence evaluating the impact of interventions to improve older adults’ practical ability to take medications [41]. Thus, the need for future evaluations and interventions on this subject proves to be highly relevant.

Recruitment site also remained independently associated with medication non-adherence, with participants recruited in Curitiba presenting higher odds of non-adherence than those recruited in Londrina. This finding should be interpreted in the context of the substantial differences in participant profiles between the two sites. Compared with Londrina, the Curitiba sample included a higher proportion of patients recruited from cardiology clinics and presented a greater number of comorbidities, higher medication burden, greater prevalence of dynapenia and functional dependence, higher loneliness scores, and a greater frequency of belief-related barriers. Although outpatient clinic specialty and other relevant characteristics were considered in the multivariable modeling process, recruitment site remained independently associated with the outcome. No statistically significant interactions were identified between recruitment site and the characteristics that differed between centers. Thus, the observed site association may reflect differences in case mix, patterns of care, or other center-specific characteristics not fully captured by the measured covariates and should not be interpreted as an intrinsic effect of geographic location.

In contrast to the significant independent association observed for recruitment site, other sociodemographic factors, such as age, sex, education, and income, were not independently associated with the outcome in the univariable analyses. This could be due to the lower social status in our whole sample.

According to the Brazilian Institute of Geography and Statistics (IBGE), the majority of Brazilian older adults have low education (less than 7 years of study), have an average family income between 1 and 3 minimum wages [42], live accompanied, and are female [43]. Thus, the external validity of the study is reinforced, as there is a convergence between population indicators and our results, demonstrating that the present study captured the structural socioeconomic disparities in the Brazilian context.

Despite being a global health problem, there is no consensus on the prevalence of medication adherence at national or global levels. This is due to adherence being a continuous process that is difficult to evaluate. The absence of a gold-standard method, combined with the existence of a vast amount of validated questionnaires to estimate adherence, hinders comparison between studies.

Beyond these extrinsic constraints, the study presents certain intrinsic limitations that persist despite the reported contributions. Due to its cross-sectional nature, we cannot establish causality. The study relied on complete-case analysis without employing imputation methods for missing data, which could introduce bias if the missingness is not completely at random. By recruiting from specialized outpatient clinics, there is a potential “healthy user bias,” participants actively attending appointments may be inherently more adherent than those who have dropped out of the system, potentially underestimating the true prevalence of non-adherence in the general older population. Also, adherence was assessed via self-report (MAT), which is subject to recall and social desirability biases, potentially leading to overestimation.

Despite these limitations, the study has notable strengths. It is a pragmatic, real-life investigation of older adults followed in tertiary outpatient clinics of the Brazilian public health system, addressing underexplored dimensions of a global phenomenon. Most importantly, to the best of our knowledge, this is the first study to describe a graded association between different levels of loneliness and medication non-adherence.

## Conclusion

Medication non-adherence during the implementation phase affects approximately 1 in 5 older adults followed in specialized public outpatient clinics and emerges as a multidimensional sociobehavioral phenomenon. Belief-related barriers, practical barriers, recruitment site and loneliness were independently associated with non-adherence, with loneliness demonstrating a graded association pattern, progressively amplifying the risk as social disconnection worsens. These factors are not isolated factors but converging dimensions of a broader biopsychosocial vulnerability that shapes how older adults engage with their pharmacological treatment over time. Interventions targeting medication non-adherence in this population could potentially move beyond pill-centered approaches to incorporate strategies that address attitudinal barriers, functional limitations, site-level contextual complexities and social isolation as interconnected and modifiable targets. Future longitudinal studies are warranted to clarify the directionality of these associations and to evaluate the effectiveness of multicomponent, person-centered interventions in improving adherence in aging populations within public health systems.

## Supporting information

S1 Table Characteristics of study participants according to recruitment site. (DOCX)

S2 Table Variance Inflation Factor (VIF) results for the assessment of multicollinearity among variables included in the multivariable model for medication non-adherence. (DOCX)
