# Supplementary material for: Loneliness and barriers to medication adherence in older adults: A multicenter cross-sectional study
Source: PLoS One. 2026 Nov 13;21(11):e0352895. doi: 10.1371/journal.pone.0352895 (PMC13766247; doi:10.1371/journal.pone.0352895)
Supplement: Supplementary file 1 [file pone.0352895.s001.docx]

**S1 Table -** Characteristics of study participants according to recruitment site

| **Variable** | **Recruitment Site** | | ***p* value** |
| --- | --- | --- | --- |
|  | **Curitiba, Paraná, Brazil *n*=387** | **Londrina, Paraná, Brazil *n*=210** |  |
| Age | 70.8 ± 6.9 | 72.2 ± 7.7 | .025^§^ |
| Male gender | 184 (47.5) | 77 (36.7) | .012* |
| Education |  |  | .023^#^ |
| Low (≤ 7 years of study) | 282 (72.9) | 174 (82.9) |  |
| Medium (8 - 11 years of study) | 70 (18.1) | 24 (11.4) |  |
| High (≥ 12 years of study) | 35 (9.0) | 12 (5.7) |  |
| Marital status |  |  | .121^#^ |
| Married or stable union | 204 (52.7) | 123 (58.6) |  |
| Widowed | 87 (22.5) | 45 (21.4) |  |
| Separated or divorced | 54 (14.0) | 31 (14.8) |  |
| Single | 42 (10.9) | 11 (5.2) |  |
| Outpatient clinics |  |  | <.001^#^ |
| Cardiology | 257 (66.4) | 44 (21.0) |  |
| Internal Medicine or Family and Community Medicine | 18 (4.7) | 14 (6.7) |  |
| Endocrinology | 60 (15.5) | 27 (12.9) |  |
| Gastroenterology | 41 (10.6) | 19 (9.0) |  |
| Others | 11 (2.8) | 106 (50.5) |  |
| Number of comorbidities | 6 (4 - 8) | 5 (3 - 7) | <.001^‡^ |
| Muscle status by sex-specific cut-offs, according to dynamometry. ^a,b^ |  |  | <.001* |
| Eutrophic | 207 (53.8) | 167 (80.7) |  |
| Dynapenia | 178 (46.2) | 40 (19.3) |  |
| Physically inactive (< 150 min/week) | 303 (78.3) | 173 (82.3) | .243* |
| Sedentary time (hours)^c^ | 4 (2 - 7) | 4 (2 - 5) | <.001^‡^ |
| Functional dependence for BADL^d^ | 132 (34.1) | 32 (15.2) | <.001* |
| Living alone | 65 (16.8) | 41 (19.5) | .433* |
| UCLA-BR loneliness score, range 0 - 60 | 9 (4 - 18) | 3 (1 - 8) | <.001^‡^ |
| Cognitive impairment, classified by education-adjusted MoCA scores | 176 (45.5) | 105 (50.0) | .304 |
| Number of pills per day | 10 (6.5 - 14) | 6 (3 - 9) | <.001^‡^ |
| Presence of belief-related barriers | 124 (32.0) | 12 (5.7) | <.001* |
| Presence of one or more practical barriers | 315 (81.4) | 157 (74.8) | .059* |

Categorical variables are expressed as absolute frequencies (percentages). Age is reported as mean ± standard deviation (SD), and other quantitative variables are expressed as median (*Md*) and interquartile range (IQR).

^a^ Handgrip strength was assessed based on three consecutive measurements in both hands. The highest value was used for statistical analysis. Values below 16 kgf for women and 27 kgf for men were defined as dynapenia.

^b^ 1 missing data point in the Curitiba site and 3 in the Londrina site.

^c^ Sedentary time was estimated based on self-reported daily sitting hours per day

^d^ The basic activities of daily living (BADLs) assessed by the Katz Index include: bathing, dressing, toileting, transferring, continence, and feeding

* Significance based on Fisher's exact test.

\# Significance based on the Chi-square test.

§ Significance based on the Student's t-test for independent samples.

‡ Significance based on the non-parametric Mann-Whitney U test.
