# Supplementary material for: Loneliness and barriers to medication adherence in older adults: A multicenter cross-sectional study
Source: PLoS One. 2026 Nov 13;21(11):e0352895. doi: 10.1371/journal.pone.0352895 (PMC13766247; doi:10.1371/journal.pone.0352895)
Supplement: Supplementary file 2 [file pone.0352895.s002.docx]

**S2 Table** - Variance Inflation Factor (VIF) results for the assessment of multicollinearity among variables included in the multivariable model for medication non-adherence

| **Independent variables included in the multivariable model for medication non-adherence** | Variance Inflation Factor (VIF) |
| --- | --- |
| Recruitment site | 1.804 |
| Outpatient clinics | 1.452 |
| Education level | 1.054 |
| Marital status | 1.015 |
| Sedentary time (hours) | 1.075 |
| History of falls in the past 12 months | 1.148 |
| Dynapenia | 1.140 |
| Functional dependence for Basic Activities of Daily Living | 1.190 |
| Brazilian version of the UCLA Loneliness Scale, range 0 - 60 | 1.201 |
| Presence of belief-related barriers | 1.138 |
| Presence of one or more practical barriers | 1.057 |
| Number of pills per day | 1.204 |
